# Do Surgical Interventions Influence Psychosexual and Cosmetic Outcomes in Women with Disorders of Sex Development?

**DOI:** 10.5402/2012/276742

**Published:** 2012-03-05

**Authors:** Nina Callens, Yvonne G. van der Zwan, Stenvert L. S. Drop, Martine Cools, Catharina M. Beerendonk, Katja P. Wolffenbuttel, Arianne B. Dessens

**Affiliations:** ^1^Division of Pediatric Endocrinology, Department of Pediatrics, Erasmus MC-Sophia, P.O. Box 2060, 3000 CB Rotterdam, The Netherlands; ^2^Division of Pediatric Endocrinology, Department of Pediatrics, Ghent University and University Hospital Ghent, De Pintelaan 185, 9000 Ghent, Belgium; ^3^Department of Obstetrics and Gynaecology, Radboud University Nijmegen Medical Center, P.O. Box 9101, 6500 HB Nijmegen, The Netherlands; ^4^Department of Urology, Erasmus MC-Sophia, P.O. Box 1738, 3000 CB Rotterdam, The Netherlands

## Abstract

Clinical practice developed to promote psychosexual well-being in DSD is under scrutiny. Although techniques for genital surgery have much improved lately, long-term studies on psychosexual functioning and cosmetic outcome on which to base treatment and counseling are scarce. We studied 91 women with a DSD. Feminizing surgery was performed in 64% of the women; in 60% of them, resurgery in puberty was needed after a single-stage procedure. Both patients and gynecologists were satisfied with the cosmetic appearance of the genitalia. However, forty percent of these females experienced sexuality-related distress and 66% was at risk for developing a sexual dysfunction, whether they had surgery or not. Recognizing the difficulty of accurate assessment, our data indicate that feminizing surgery does not seem to improve nor hamper psychosexual outcome, especially in patients with severe virilization.

## 1. Introduction

Disruption of genetic pathways involving a complex network of genes in the developing embryo may lead to disorders of sex development (DSD). These are congenital conditions with atypical development of chromosomal, anatomical, or gonadal sex [1]. Depending on the diagnosis in infants with a female gender assignment, medical corrective measures can include hormone replacement therapy and the removal of gonads in addition to surgical adjustment of the external genitalia. Genital surgery can range from the construction of a vagina or enlarging of the vaginal opening, to clitoris reduction, and adjustment of the labia [2]. The primary aim of surgery is to provide a female appearance of the (masculinized) genitalia and enabling tampon use and sexual intercourse later in adulthood. In a minority of patients, it is indicated to promote bladder emptying and to avoid urological infections.

The medical and psychological management of DSD has long been interrelated, since medical interventions have attempted to promote “successful” psychological adjustment to the established sex and corresponding gender [3]. It is presumed that “normalizing” the bodies of individuals with a DSD will be beneficial for psychosocial development and will avoid stigmatization. Advances in medical knowledge and understanding of psychosexual development, together with an increased emphasis on the rights of the individual and changing attitude towards gender, have all contributed recently to the debate about this “success” and efficacy of medical intervention [4]. The advent of well-organized support groups has given patients the confidence to express their concerns, and there is increasing evidence of dissatisfaction with surgery [5], relating to both the timing and outcome following surgery [6–12]. Genital surgery may have created sexual dysfunctions (including altered genital sensation) and/or dissatisfaction with cosmetic outcomes [5, 13]. It can be questioned that the very approach that was adopted to prevent psychological maladjustment to DSD is in fact the cause of the high levels of psychological and sexual distress reported [14].

While some surgeons are recommending greater caution with the surgical approach [15] and critics point out that the current treatment practice exposes individuals with DSD to multiple traumatization [9], others remain convinced that children would benefit from having genital surgery, particularly girls with congenital adrenal hyperplasia [3].

This study was undertaken to inform the debate about the role of genital surgery in the treatment of DSD and to create a better understanding of the factors that support psychosexual adjustment across the lifespan. Our objective was to evaluate the influence of genital surgery on psychosexual and cosmetic outcome in association with the specific DSD etiology and degree of virilization at birth, for adjusting our clinical services accordingly to provide the best possible care.

## 2. Methods

### 2.1. Procedure and Patients

The study was conducted as a long-term follow-up audit of DSD patients referred to Erasmus Medical Centre Rotterdam or Radboud University Nijmegen Medical Center, the Netherlands, between the years 2007–2010. 412 patients with a DSD were identified from the hospital databases. After exclusion, 176 eligible participants were contacted, inviting them to attend a clinical visit with a gynecologist and psychologist. Exclusion criteria were age younger than 14 years and over 60 years, intellectual disability, recent diagnosis (<6 months), a diagnosis of Mayer-Rokitansky-Küster-Hauser (MRKH), Turner syndrome or Klinefelter syndrome, and anatomical anomalies of the abdomen not related to a deviant gonadal development such as cloacal malformations. 91 patients (52%) participated. Patient population, diagnosis information, and details of the received surgical treatments are summarized in Tables 1 and 2. Diagnostic subgroups were classified according to the consensus statement classification [1]. The median age was 26 years (range 14–48). All patients gave written informed consent, and the study was approved by the medical ethics committee of both institutions.

### 2.2. Assessments

#### 2.2.1. Gynecological Assessment

 The gynecological workup consisted of a (1) medical-somatic history and (2) a gynecological exam: visual inspection (clitoral size, labia majora, labia minora, pigmentation, meatus externus urethrae, hair growth, labial scarring, perineum length), speculum examination (assessment of the vagina, internal hair growth, granulated tissue, epithelial atrophy, presence of a cervix), and pelvic examination (accessibility by number of fingers, vagina length and width (Hegar), strictures, pelvic floor tone, vaginal discharge). In addition, cosmetic scores (scale 1–10; 1 = extremely poor, 10 = excellent, <6 was insufficient) were given by both the gynecologists and patients. The gynecologists in both institutions had not been previously involved in the gynecological care of these women.

Data on genital surgeries as well as the degree of virilization at birth were retrospectively collected from the medical files. The degree of genital masculinization in females with congenital adrenal hyperplasia (CAH) is typically described according to Prader stages, but in a number of our patients the Prader stage was not recorded. More recently, the external masculinisation score (EMS) was developed to assess the degree of undervirilization in an individual with 46, XY DSD. The score is based on the presence or absence of a micropenis and bifid scrotum, the location of the urethral meatus and the position of the testes [16]. For patients with gonadal dysgenesis, however, no such classification system has been developed (yet). Therefore, we developed a categorization system that could be applied to the entire group of patients: (1) Patients with 46, XY DSD and Chromosomal (Chrom) DSD with normal appearing female external genitalia (46 XY, NF) (i.e., patients with a normally sized clitoris, normally developed labia minora and majora, vaginal dysplasia and gonads in the abdomen or groins (2); patients with 46, XY DSD and Chromosomal DSD with various degrees of virilization of the external genitalia (46, XY AG) (i.e., patients with an enlarged clitoris, partially or completely fused labia, vaginal dysplasia and gonads in the abdomen or groins (3); patients with 46, XX DSD with various degrees of virilization of the external genitalia (46, XX AG) (i.e., patients with congenital adrenal hyperplasia (CAH)) often born with ambiguous genitalia such as an enlarged clitoris, partially or completely fused labia, small introitus, or confluence of the vagina and urethra (see Table 1).

#### 2.2.2. Psychological Assessment

A sexual dysfunction, according to the DSM-IV-TR, is diagnosed by decreased sexual function and increased sexual distress. Sexual functioning was assessed by the Female Sexual Function Index (FSFI) questionnaire [17], validated for the Dutch speaking population [18]. This short 19-item quiz assesses adult female sexual quality of life in the 4-week period before completing the survey and its score is unbiased regarding age, education, and economic status. The 19 items are assigned to six separate domains of female sexual function: desire, arousal, orgasm, sexual pain, vaginal lubrication, and global sexual and relationship satisfaction. Since women without partner were not able to answer items 14 and 15 (relating to relationship satisfaction), their satisfaction score was solely based on item 16 (relating to global sexual satisfaction). An adjusted total FSFI score was calculated as well. Sexual distress was assessed by the Female Sexual Distress Scale-Revised (FSDS-R) [19]. Additionally, a semistructured interview provided in-depth information about psychosexual and social adjustment. Clinical psychologists in each centre performed the psychosexual assessments; they had not been involved in the care of these women.

#### 2.2.3. Statistics

In order to evaluate the cosmetic outcome, univariate and backwards stepwise linear regression analyses were used to identify factors associated with cosmetic outcome. The relationship between continuous parameters and phenotype/virilization or surgical procedure was analyzed using the Spearman's rank order coefficient. Confounding and effect modification were assessed with stratified backwards stepwise linear regression analysis. We looked for a significant contribution of the interaction effects on the predictive ability of the model by adding the interaction effects to the main effects.

Psychosexual functioning in relation to genital virilization and genital surgery was assessed by an ANCOVA with psychosexual outcomes as the dependent variables, genital surgery (yes/no) and category of virilization as fixed factors, and number of genital surgeries and age at first surgery entered as covariates to correct for confounding variables. Categorical data were analyzed using the *χ* ^2^ test or Fisher's exact test. The SPSS 17.0 software was used for all statistical calculations. A *P* value <0.05 was considered statistically significant. Two-tailed statistical tests were chosen to reduce the risk of type I errors. 

## 3. Results

Surgery (vaginoplasty and/or clitoroplasty) was performed in 58/91 (64%) of the women. Details are given in Table 2.

Using the classification based on degree of virilization at birth, 36/40 (90%) of the patients with 46 XX DSD (i.e., CAH) had feminizing surgery. Three out of the 4 patients who did not have surgery had the simple virilizing form. Seven out of 30 (23%) of the patients with 46, XY/Chrom DSD, and a normal female appearance of the external genitalia had a vaginoplasty (4/15 with CAIS, 3/7 with complete gonadal dysgenesis). Two of the women with complete GD had a single-stage surgery (i.e., vaginoplasty and clitoroplasty). 15/21 (71%) of the patients with 46, XY/Chrom DSD, and ambiguous external genitalia received feminizing surgery (9/13 patients with PAIS, 17*β*3 hydroxysteroid dehydrogenase deficiency, or 17, 20 lyase deficiency and 6/8 with partial gonadal dysgenesis). The mean age of first surgery was significantly lower in the group of females with 46, XX DSD and ambiguous external genitalia (M = 4.1 years, range 0–19 years) than in the group of females with 46, XY/Chrom DSD, and ambiguous external genitalia (M = 12.9 years, range 0–27 years) or group of females with 46, XY/Chrom DSD with a normal female appearance of the external genitalia (M = 17 years, range 0–28 years) (*P* = 0.001). The total number of genital surgeries was also higher in the group of females with 46, XX DSD and ambiguous external genitalia (median 2, range 0–4), than the group with 46, XY/Chrom DSD and ambiguous external genitalia (median 1, range 0–4) or 46, XY/Chrom DSD NF (median 0, range 0–2) (*P* < 0.001). 25/91 patients had undergone a clitoroplasty in childhood, of them 10 underwent a vaginoplasty and 11 an introitusplasty in puberty. Another 20 patients had undergone a genitoplasty planned as single stage. 7 of them needed a revaginoplasty and 5 a Reintroitusplasty because of vaginal insufficiency (see Table 2). 30/51 women who had a vaginoplasty (with or without concurrent clitoroplasty) had to follow a home routine dilation programme after surgery to maintain their vaginal length or introitus width (when having no regular sexual intercourse). 14/51 women did not have to do this, and in 7 women this information was missing. Of the 33/91 women who had no feminizing surgery, 9 women followed a vaginal dilation programme to create sufficient vaginal length. The other 24 women had no medical genital treatment.

Eight women (17%) of the women experienced some urinary incontinence (urge, stress, or both), of them seven had undergone surgery, and one had not.

### 3.1. Cosmetic Outcome

A third of the patients refused to take part in the gynecological checkup. Reasons for refusal were related to time pressure but more often having undergone examinations of the genitalia that had been experienced as shameful.

The cosmetic outcomes scores, given by both patients and gynecologists, are summarized in Table 3. Gynecologists scored the appearance of the genitalia on average higher than the patients. For the clitoris and labia majora, scores of patients and gynecologists did not differ (*P* > 0.05); for the labia minora scores were different (*P* = 0.013). No comparison for the vagina could be made, since only gynecologists rated this aspect of the genitalia.

A satisfactory cosmetic outcome, defined as a mean gynecologic score above 6, was present in all the diagnostic groups (M = 7.4, sd = 1.4). Women who had undergone surgery had on average lower cosmetic scores than women who did not have any surgery (M = 7.0, sd = 1.25 versus M = 8.2, sd = 1.37) (*P* = 0.000) (Table 4), but this difference disappeared when corrected for the virilization degree at birth, total number of surgeries, and age at first surgery (*P* = 0.073, ns). Age at first surgery and total numbers of surgeries were significantly associated with the cosmetic outcome score (*r* = 0.421, *P* = 0.005 and *r* = −0.441, *P* < 0.001, resp.), indicating that a lower cosmetic outcome was associated with a younger age at first surgery and a higher number of undergone surgeries. Regression analysis showed that only the level of virilization had a significant effect on the cosmetic outcome score (*P* = 0.022), after correction for number of surgeries and the interaction effects of surgery with virilization. The adjusted *R* ^2^ of this model was 0.217. 

To see whether age at first surgery contributed to the cosmetic outcome score we performed a regression analysis on the group that was operated. After correction for number of surgeries, degree of virilization, and the interaction effects of age at first surgery and degree of virilization, there was no significant contribution of the age at first surgery on the cosmetic outcome (*P* = 0.113). The adjusted *R* ^2^ of this model was 0.173.

### 3.2. Psychosexual Outcome

8 women did not want to fill in the FSFI because they did not want to answer in details questions about their sexuality. Of the other 83 women, 25 women (30%) were not sexually active in the 4-week period before taking part in the study. A variety of reasons was reported, ranging from having no time or no need for sex, to a problematic confrontation with the body and diagnosis. An extra 15 women specifically did not have sexual intercourse. In fact, 41% (32/79) never had sexual intercourse at the moment of follow-up (10 women did not have had surgery, 22 had had surgery). Those who never had sexual intercourse were on average younger (median 18 years, range 14–46 years) than those who had intercourse (median 23 years, range 19–48 years) (*P* = 0.034). The in-depth interview revealed that reasons for virginity mostly related to age (i.e., considering themselves too young), sexual orientation (some women with CAH and PAIS only had sexual experiences in lesbian relationships), and considering their bodies unsuitable for having intercourse (i.e., vagina was too narrow, although this was anatomically not confirmed).

The original total FSFI score was only known in 35 women, because of the missing values on the domains of satisfaction and pain. 40% (36/85, in 6 women this information was missing) of the women had no (sexual) partner. 66% of the women (23/35) with a total FSFI score scored below the clinical cut-off of 26.55 on the FSFI, which indicates that they are at risk for developing a sexual dysfunction.

An adjusted total FSFI score was calculated because of the above-mentioned drawbacks with the FSFI. In 22 patients with no partner, the satisfaction domain was solely based on one item. In the other patients without partner, no data on satisfaction were available and the FSFI score could not be calculated for them. This problem was reported before by Meyer-Bahlburg and Dolezal [20]. A similar problem was seen with items in the pain domain, resulting in a pain score based on 2 items instead of 3. An adjusted FSFI score, with only adjusted satisfaction and pain scores, was then known in 60/91 patients. 

The total FSDS-R score was known in 76 women. In 41% (31/76) of these women, the FSDS score is above the clinical cut-off score of 11, indicating that they experience sexuality-related distress.

**Figure 1:**
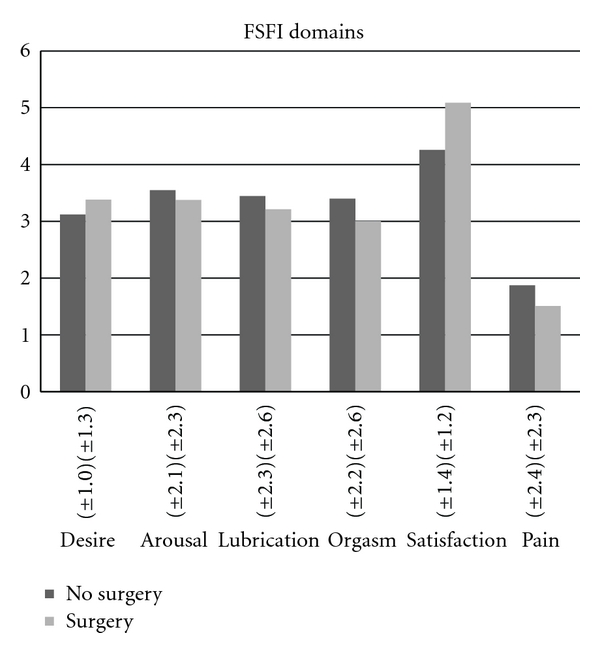
Mean scores (±sd) on the six domains of the Female Sexual Function Index (0–6) in women with and without feminizing surgery.

No significant differences were found between women with and without surgery on the total FSFI, adjusted FSFI, and FSDS (Table 4), nor on the different FSFI domains, expect for the satisfaction domain (Figure 1). Women who had surgery were more satisfied with their sexual life than women without surgery (*P* = 0.037). However, when the adjusted satisfaction score was used, this difference disappeared. No significant differences were found between women who had surgery and those who had not on the total FSFI (original, adjusted) (*P* = 0.395 and *P* = 0.455, ns), nor on the different FSFI domains when corrected for the number of surgeries, level of virilization, and age at first surgery. Also no significant difference was found when the same model was tested with the FSDS-R as dependent variable (*P* = 0.078, ns). No significant associations were found between age at first surgery, total number of surgeries, and virilization degree on the one hand and total FSFI and FSDS on the other hand.

## 4. Discussion

Clinical practice developed to promote psychological and sexual health in DSD is the subject of current critical review. The role of genital surgery, in particular, is under intense scrutiny. Crucial to the debate on the role of surgery are conflicting empirical long-term outcome data.

The results of this study show that, while cosmetic outcomes are sufficient, a large proportion of the patients suffer from psychosexual difficulties. 40% experienced sexuality-related distress and 66% of the sample was at risk for developing a sexual dysfunction. However, no major influence of genital surgery on cosmetic and psychosexual outcomes was found.

Recognizing the difficulty of accurate assessment, our study suggests that surgery does not seem to improve sexual satisfaction. On the other hand, there is no evidence that feminizing surgery has worsened psychosocial outcomes. Those patients with the highest degrees of genital ambiguity might actually have benefitted from surgery, as their scores on sexual functioning were at the same level as those women with DSD with less severe virilization of the external genitalia. This observation, however, is not in line with those found in previous studies [21].

There are isolated reports of successful psychosexual outcome in adult females with ambiguous genitalia, but their numbers are unclear and their case histories have not been well documented [22]. Although heavily influenced by cultural and ethnic issues, observations made by Warne (not published) in India and Vietnam and our own observations in Indonesia suggest that most individuals who grow up with ambiguous genitalia—because surgery was not available—are suffering from social stigmatization on a daily basis.

However, altered genital sensation and inability to achieve orgasm were reported after clitoroplasty in some series [13, 21, 23–25], even with the use of modern surgical techniques to preserve clitoral sensation, but not in others [26]. While older studies, looking at psychosexual function after vaginoplasty, reported reasonable outcomes [27], subsequent studies have shown both positive and negative effects on sexual development and activity [28]. Some reported a high incidence of sexual dysfunction, especially in CAH women with higher virilization grades [7, 21, 29]. Although long-term cosmetic outcomes are average to good (assessed by gynecologists), resurgery for complications such as vaginal stenosis or fistula's are common [30]. Creighton et al. [31] found that 98% needed further treatment of the vagina for tampon use or intercourse. This was also confirmed in our study, where in 60% resurgery in puberty was needed after an initial single-stage procedure because of a narrow introitus and/or for more extensive reconstruction because of vaginal insufficiency.

When feminizing surgery is performed in cases of an absent or short vagina, such as in complete androgen insensitivity syndrome, the first line of treatment should be vaginal dilators [32, 33] due to the absence of surgical risk and preservation of vaginal tissue. In the past, surgical methods were the cornerstone of treatment, although postoperative vaginal dilation still had to be performed. In this sample, 68% of the women had to dilate after surgery. Available studies on primary vaginal dilation programmes claim success rates in the region of 80% [34–36]. 9 out of 91 women in our sample followed a primary vaginal dilation programme. Since this group was rather small, no definite conclusions could be reached in terms of sexual functioning, especially compared to vaginal surgery. Most studies investigating sexual function after (different procedures of) vaginoplasty, however, agree that women with vaginal hypo- or aplasia are at risk for developing sexual problems (difficult lubrication, inability to experience orgasm, and dyspareunia), despite treatment [37–41], with both psychological and physical factors probably predisposing for sexual difficulties.

Currently most clinicians consider that childhood genital surgery is indicated in those with a higher degree of genital ambiguity to avoid the assumed psychological distress of passing through childhood and adolescence with abnormal looking genitalia [1]. However, it has been suggested that a vagina is not necessary for a young girl prior to menarche or sexual intercourse. This was confirmed by women with complete absence of the vagina (e.g., CAIS), who indicated no psychological or developmental problems until they experienced primary amenorrhea. It would seem logical then to defer vaginal surgery until later in life (although this is explicitly not in agreement with the consensus statement [1]). This should also limit the total number of operations an individual will undergo and allow for the patient to consent and to be involved in the decision for surgery [5]. Moreover, since the risk of stenosis is high after prepubertal vaginoplasty and moderate clitoral hypertrophy may regress with medical therapy for CAH, other surgery such as vaginoplasty may be delayed [42]. On the other hand, when a clitoroplasty is indicated for severe clitoral hypertrophy, the redundant clitoral skin can be used for a vaginoplasty in the same procedure. 

Our results did not indicate that the number of surgeries performed or the age at first surgery were associated with a better cosmetic outcome. As was also shown in our study and highlighted by Alizai et al. [28], total correction cannot always be achieved with a single operation in infancy. On the other hand, while the evidence that feminizing surgery will improve psychological outcomes is lacking, there are objective reasons why patients being treated with genital surgery now should expect a better outcome than patients treated 20–30 years ago. Clitoral surgery has improved, in addition to counseling methods [22]. It is becoming increasingly clear that one of the most important factors determining the success of operations such as vaginoplasty is the psychological coping of the patient [43]. 

A large proportion of the women in the semistructured interview mentioned the struggles to cope with their diagnosis, reinforced by societies' ignorance of DSD conditions and general taboos over sexual issues. Some of them described the loss of a sense of normality and equality with peers and the impossibility of communication about it. Especially the distress caused by infertility seemed to leave some of these women inferior to others. This self-perception may negatively affect a person's sexual esteem and behavior and lead to sexual difficulties [8]. Another explanation for the high degree of psychosexual distress and dysfunction reported is a sort of self-fulfilling prophecy, where the women might have felt that sexual difficulties were to be expected and reported accordingly. In contrast, satisfaction levels were not very low, indicating that perhaps the women were not dissatisfied with the low expectations of sexual functioning, precisely because they were to be expected [8]. 

We recognize several weaknesses of this study. Firstly, it was difficult to precisely quantify the degree of virilization due to the heterogeneity of DSD diagnoses, and thus different underlying virilization mechanisms. A variety of techniques had to be used to assess the virilization degree and the classification system we used has not been described before. Secondly, there was a potential selection bias, since participants in this study were recruited exclusively from a clinical sample. Possibly, women who opted into the study were those who had reached a stage of acceptance and felt able to talk about their experiences. Alternatively, clinically recruited patients may have a higher incidence of complications, including sexual difficulties, and a call to take part in a study may have prompted them to seek medical advice [8]. Further studies should also recruit from other samples, such as peer-support groups. Additionally, data on nonresponders should be gathered. Half of the women who have been tracked refused to participate, indicating that physical aspects of female sexuality are still a very sensitive subject. Secondly, we were unable sufficiently to check some factors other than surgery that may affect sexual function. Other presumed factors such as the social stigma attached to having a DSD, diagnostic secrecy practiced by some clinicians, and other unresolved psychological issues and anxieties related to living with this condition need to be further explored. Lastly, this was a retrospective follow-up study and no information was available on psychosexual and social functioning before treatment. Prospective, longitudinal studies with a focus on diagnosis-related success rates should be undertaken.

## 5. Conclusions

Feminizing genitoplasty procedures have changed dramatically since the 1980s, but still there is no consensus and there are few long-term data on which to base the decision of the timing on genital surgery. One of the major problems is our knowledge of the parameters determining “sexual quality of life.” Endocrine factors are probably as important as the genetic factors, the environmental, cultural, and educational ones, to establish a sexual profile [44]. Every woman with DSD should, therefore, be referred to a multidisciplinary team, integrating endocrinology, sexology, and gynecology expertise. Clinical psychological support should be available in an ongoing matter into adulthood, as psychological aspects of living with a DSD condition impact in many areas of life. Any surgery offered, including vaginoplasty, clitoral surgery, and gonadectomy should be presented with full information on both the potential benefits and the risks of the procedure.

## Figures and Tables

**Table 1:** Patient population.

		Virilization degree			Total
		46 XY/Chrom DSD NF	46 XY/Chrom DSD AG	46 XX DSD AG	
	CAH SW	0	0	32	32
	CAH SV	0	0	8	8
	Undervirilization-Some androgen action*¹*	0	13	0	13
DSD group	Undervirilization-CAIS	19	0	0	19
	Gonadal dysgenesis-46, XY	7	0	0	7
	Gonadal dysgenesis-chromosomal	4	8	0	12

Total		30	21	40	91

*¹*Partial androgen insensitivity syndrome (PAIS) [5], 17*β*3 hydroxysteroid dehydrogenase deficiency (17*β*3 HSD) [5], Leydig cell hypoplasia [2], 17, 20 lyase deficiency [1].

**Table 2:** Number of clitoroplasties and vaginoplasties performed in the different etiological groups.

		Clitoroplasty	Vaginoplasty	No surgery
46 XX DSD AG		37	32	4
	CAH-SW	30	29	1
	CAH-SV	5	3	3

46 XY/Chrom DSD NF		2	7	23
	46 XY GD	2	3	4
	Chromosomal GD	0	0	4
	No androgen action (CAIS)	0	4	15

46 XY/Chrom DSD AG		10	11	6
	Some androgen action	4	6	4
	Chromosomal GD	6	5	2

**Table 3:** Cosmetic outcome score [1–10] based on four aspects of the external genitalia.

		Mean	Sd
Clitoris (*n* = 63)	Gynecologist	7.16	1.94
	Patient	6.76	1.89
Labia majora (*n* = 66)	Gynecologist	7.65	1.60
	Patient	7.11	1.48
Labia minora (*n* = 64)	Gynecologist	7.28	1.97
	Patient	6.73	1.81
Vagina (*n* = 60)	Gynecologist	7.32	1.79

**Table 4:** Univariate analysis of cosmetic and psychosexual outcomes in the surgery—no surgery group. Only cosmetic outcome was significantly different in the surgery versus non surgery group (*P* < 0.001).

	Cosmetic outcome			FSDS			FSFI			Adjusted FSFI		
SURGERY	Mean	Sd	*N*	Mean	Sd	*N*	Mean	Sd	*N*	Mean	Sd	*N*
No surgery	8.21	1,37	25	10.23	10,4	25	20.71	10,16	15	21.67	9.35	23
Surgery	6.97	1,25	45	11.00	9,62	51	22.17	9,4	20	20.95	10.1	41
